# Individualizing the dosage of *Methylphenidate* in children with attention deficit hyperactivity disorder

**DOI:** 10.1186/s12874-020-00934-y

**Published:** 2020-03-11

**Authors:** Hoda Shirafkan, Javad Mahmoudi-Gharaei, Akbar Fotouhi, Seyyed Ali Mozaffarpur, Mehdi Yaseri, Mostafa Hoseini

**Affiliations:** 1 Social Determinants of Health (SDH) Research Centre, Research Institute for Health, Babol University of Medical Sciences, Babol, Iran; 2 Department of Epidemiology and Biostatistics, School of public health, Tehran University of Medical Sciences, Tehran, Iran; 3 Psychiatry and Psychology Research Center, Roozbeh Hospital, Tehran University of Medical Sciences, Tehran, Iran; 4 Traditional Medicine and History of Medical Sciences Research Center, Health Research Institute, Babol University of Medical Sciences, Babol, Iran

**Keywords:** Precision medicine, Tailor medicine, Attention deficit disorder with hyperactivity, Methylphenidate, Mixed-effect models, Longitudinal data

## Abstract

**Background:**

Attention deficit hyperactivity disorder (ADHD) is one of the most common childhood mental health disorders. Stimulant drugs as the most commonly used treatment and first-line therapy for ADHD have side effects. One of the newest approaches to select the best choices and optimize dosages of medications is personalized medicine.

**Methods:**

This historical cohort study was carried out on the data taken from the period of 2008 to 2015. Eligible subjects were included in the study randomly. We used mixed-effects logistic regression models to personalize the dosage of Methylphenidate (MPH) in ADHD. The patients’ heterogeneity was considered using subject-specific random effects, which are treated as the realizations of a stochastic process. To recommend a personalized dosage for a new patient, a two-step procedure was proposed. In the first step, we obtained estimates for population parameters. In the second step, the dosage of the drug for a new patient was updated at each follow-up.

**Results:**

Of the 221 children enrolled in the study, 169 (76.5%) were male and 52 (23.5%) were females. The overall mean age at the beginning of the study is 82.5 (± 26.5) months. In multivariable mixed logit model, three variables (severity of ADHD, time duration receiving MPH, and dosage of MPH) had a significant relationship with improvement. Based on this model the personalized dosage of MPH was obtained.

**Conclusions:**

To determine the dosage of MPH for a new patient, the more the severity of baseline is, the more of an initial dose is required. To recommend the dose in the next times, first, the estimation of random coefficient should be updated. The optimum dose increased when the severity of ADHD increased. Also, the results show that the optimum dose of MPH as one proceeds through the period of treatment will decreased.

## Background

Attention deficit hyperactivity disorder (ADHD) is one of the most common childhood mental health disorders [1]. The estimated prevalence of ADHD in Iranian children and adolescents is ranging between 2.8 and 19.9% [2] and ranging from 5 to 12% in school-aged children worldwide [3].

The etiology of ADHD is multi-factorial [4]. It is a combination of genetic [5, 6] and environmental (e.g., exposure to alcohol or lead, prenatal maternal smoking, prematurity, pregnancy complications, and low birth weight) [7, 8] factors. ADHD symptoms (hyperactivity, impulsiveness, and a developmental lack of attention) could cause significant damage in school tasks [9] and in the functions of daily activities [10]. In most children with ADHD, symptoms continue into adolescence and adulthood; it leads to social, occupational and personal dysfunctions [11]; therefore, early diagnosis and appropriate treatment could be beneficial [12]. ADHD often accompany other mental and behavioral disorders [9].

Stimulant drugs as the most commonly used treatment and first-line therapy for ADHD have side effects including abdominal pain, nausea, loss of appetite, nervousness, insomnia, compulsive behaviors and movement disorders [13]. Unclear long-term benefits due to undesirable side effects of psychopharmacological treatments, caused scientific society to search for alternative approaches to its treatment [14].

One of the newest approaches to select the best choices and optimize dosages of medications is personalized medicine (PM). PM tries to enable patients to receive an earlier diagnosis and optimal treatments with the least complications and the lowest costs [15]. In PM care, the genetic profile and other information of patients including concurrent medication, allergies, comorbidity, etc., are considered to establish the patient’s unique characteristics to tailor the best diagnosis and treatment [15–17].

This study evaluates the relationship between dosage of methylphenidate (MPH) and other important factors with response (improvement) in ADHD patients under the framework of a mixed-effect logit model; then proposes an optimal dose on the basis of the individualized factors of each patient.

## Methods

### Study design

This historical cohort study was carried out on the data taken from the period of 2008 to 2015 on Children with a diagnosis of ADHD who were admitted in the psychiatric clinic as a referral center and a main pediatrics hospital in Iran (the Children’s Medical Center in Tehran). The Ethics Committee of the Tehran University of Medical Sciences approved this study. The researchers followed the principles of the Helsinki Declaration.

### Participants

The children with the primary diagnosis of ADHD (based on DSM-IVTR [18] and DSM-V [19]) and the following criteria were entered to the study

1. Being within the age range of 3 to 13 years,
2. Filling the questionnaire for scaling the severity of ADHD (Conners’ Parent Rating Scale-revised Short Form (CPRS-R:S)),
3. Having at least one follow-up visit.

The exclusion criteria were as follows:

1. The main diagnosis was another disorder other than ADHD,
2. The children had just one visit (without any follow up),
3. Other drugs instead of MPH were prescribed,
4. The children didn’t respond to the treatment up to the last available follow up.(i.e. the MPH have not had any effect on them during the study time)

### Data collection

Of about 5000 available records, based on eligibility criteria, patients’ records were assessed randomly. 40% of the reviewed records did not satisfy inclusion criteria. The sampling was completed when the sample size reached 221.

### Study variables

Gender, birth weight, the age of the first diagnosis, severity of ADHD at baseline, weight per visit, type of comorbidity (if present), time intervals of visits (comparing with the first visit), dose of MPH, consumption of risperidone and fluoxetine were recorded as the basic data of the participants.

To Evaluate and score the severity of ADHD, a reliable Persian version (Cronbach alpha = 0.73) [20] of Conners’ Parent Rating Scale-revised Short Form (CPRS-R:S) was used. This scale consisted of 27 items rated from 0 (never) to 3 (very often) [21]. The mean score of at least 1.5 (i.e. crude total score greater than 40) was considered as ADHD. In each follow-up, based on this scale, diagnosis of ADHD was considered as a binary outcome variable (0 = meeting the criteria of ADHD (score > 40), 1 = not meeting the criteria of ADHD (score ≤40)). It should be declared that not meeting the criteria of ADHD (score ≤40) is the therapeutic target.

A milligram per kilogram scale was used to document the dosage of MPH.

### Statistical analysis

The basic characteristics, for quantitative variables, were summarized by mean (±standard deviation), and for qualitative variables, by frequency (percentage). The comparison of the explanatory variables between the two genders was assessed by t-test and nonparametric tests of Mann-Whitney U and chi-square. The significance level was set to 5% for all tests. The statistical analysis was carried out using R version 3.5.1 (package mle4) and STATA version 14.

### Multilevel mixed-effect logit model

We applied a generalized linear mixed model with the binomial response and a logit link,

1 $$ \log \left(\frac{P\left({Y}_{ij}=1\right)}{1-P\left({Y}_{ij}=1\right)}\right)={\beta}_0+{X}_{ij}\boldsymbol{\beta} +{b}_i+d\log\ \left({D}_{ij}\right) $$

for i = 1,…, N and j = 1,…, n_i_, where Y_ij_ indicates the binary response variable, the subscript i denoted the study subject. Here *p*_0_ will symbolize a target value for P(Y_ij_ = 1). D_ij_ is the drug dosage administered for i-th subject and j-th time point, and d is the corresponding fixed coefficient. In this model X_ij_ is the vector of covariates corresponding to fixed-effects parameter vector ***β***, *β*_0_ is a fixed intercept and *b*_***i***_ is a random-effect coefficient for the subject i. we assume it has N(0, *σ*^2^).

The vector of covariates includes gender, age of the onset of ADHD, severity of disease at baseline, birth weight, weight in follow-ups, time interval (the time passed after the commencement of treatment), taking risperidone, taking fluoxetine, being accompanied with affective disorders (mood and bipolar disorders), anxiety disorders (generalized anxiety disorder (GAD), social anxiety disorder (SAD), obsessive-compulsive disorder (OCD), phobia, and anxiety disorder), oppositional defiant disorder (ODD) and, other comorbidities (mental retardation (MR), learning disorder (LD), stutter, Tic, and major depressive disorder (MDD)). The adjusted regression coefficients (*β* = ln(*OR*)) and their 95% confidence intervals (CIs) were calculated in Table 3.

To recommend drug dosage for a new patient, a two-step procedure was proposed.

In the first step, we obtained estimates for population parameters $\hat{\boldsymbol{\beta}}$, β_0_ and $\hat{d}$.

In the second step, for the new patient *k* the estimation for $\hat{b}$_k_ was updated at each time as below.

1. At time t_1_, set $\hat{b}$_k,0_ = 0 and make the initial dose

2 $$ {D}_{k1}=\exp \left\{\frac{\mathrm{logit}\left({p}_0\right)-{\beta}_0-{X}_{k1}\hat{\boldsymbol{\beta}}-{\hat{b}}_{k,0}}{\hat{d}}\right\} $$

where the *p*_0_ is calculated using by predicting P(Y_ij_ = 1). In order to predict P(Y_ij_ = 1), we used all available responses, and then the 90th percentile of these probabilities was determined.

2. For time t_n_ (*n* > 1), based on the values of the covariates of the previous time of this individual the estimate ${\hat{b}}_{k,n-1}$ was obtained, the proposed dose for time t_n_ was obtained from the following equation

3 $$ {D}_{kn}=\exp \left\{\frac{\mathrm{logit}\left({p}_0\right)-{\hat{\beta}}_0-{X}_{kn}\hat{\boldsymbol{\beta}}-{\hat{b}}_{k,n-1}}{\hat{d}}\right\} $$

in which *b*_*i*_ is predicted by the adaptive Gauss-Hermite approximation to the log-likelihood.

## Results

Evaluating 298 records, the sample size of 221 was achieved that means 77 records didn’t meet the eligibility criteria.

This 77 records were excluded because CPRS-R:S was not correctly completed in 22 records, the main complaint was not ADHD in 19 records (five ODD, three MR, three communication disorder, two GAD, two LD, one SAD, one autism, one MDD, and one tic), other drugs were prescribed instead of MPH for 16 records, 14 records did not have any follow up, and for 6 records the age was less than 3 years old.

Of the 221 children enrolled in the study, 169 (76.47%) were male and 52 (23.53%) were female. The basic characteristics of patients between two sexes are not significantly different. The basic characteristics of these individuals are shown in Table 1.

**Table 1:** The basic characteristics of children with ADHD

Variable	Overall (*n* = 221)	Female (*n* = 52)	Male (*n* = 169)	*p*-value
Age at baseline (month; mean ± SD)	82.53 ± 26.47	85.65 ± 28.22	81.57 ± 25.92	0.332
Birth weight (kg; mean ± SD)	3.09 ± 0.56	3.07 ± 0.57	3.09 ± 0.56	0.815
Low birth weight^a^, n (%)	36 (16.3%)	7 (13.5%)	29 (17.2%)	0.528
Weight at baseline (kg; mean ± SD)	30.95 ± 5.62	25.65 ± 9.66	24.97 ± 7.28	0.191
Period of treatment (month; mean ± SD)	22.42 ± 15.19	20.13 ± 14.46	23.12 ± 15.38	0.216
Severity of ADHD at baseline (mean ± SD)	51.38 ± 10.82	48.96 ± 9.03	52.12 ± 11.23	0.065
Number of follow-ups (mean ± SD)	4.94 ± 2.83	4.69 ± 2.65	5.01 ± 2.89	0.478

^a^Children with a birth weight of lower than 2500 g were considered as having a low birth weight

In this study, six children had attention deficit disorder (ADD) (two females and four males) and 143 children had comorbidities with ADHD (55 anxiety disorders, 43 ODD, 6 affective disorders, and 39 had other comorbidities). These comorbidities were found to be as follows: anxiety disorder (GAD: 33 (14.93%), SAD: 18 (8.14%), Phobia: 6 (2.71%), OCD: 8 (3.62%), Anxiety: 7 (3.17%)); affective disorder (Mood: 5 (2.26%), Bipolar: 1 (0.45%)); Mental retardation: 4 (1.81%); Learning disorder: 20 (9.05%); TIC: 11 (4.98%); MDD: 3 (1.36%); Stuttery: 1 (0.45%); ODD: 43 (19.45%).

The overall improvement rate was calculated to be 62.70%, and no significant difference (*p* = 0.251) was observed between males (61.63%) and females (65.77%).

In the univariable mixed-logit analysis, four variables had significant relations with improvement (not meeting criteria of ADHD) including the weight of the patients in each follow-up, the severity of ADHD at the baseline, time interval of receiving the MPH, and dosage of MPH. These variables were imported to the multivariable mixed-logit model. As a result of the analysis based on this model, three variables (except weight) had a significant relationship with improvement. (Table 2).

**Table 2:** Association between explanatory variables and improvement of ADHD

		Univariable Analysis		Multivariable Analysis	
Fixed effects	Covariates	OR (95% CI)	P-Value	OR (95% CI)	*P*-Value
	Sex (Male)	0.8358 (0.5684, 1.2289)	0.361	–	–
	Age (month)	0.9991 (0.9829, 1.0053)	0.795	–	–
	Weight (kg)	1.0270 (1.0084, 1.0459)	0.004*	–	–
	Birthweight (kg)	0.8265 (0.6149, 1.1109)	0.212	–	–
	Time interval (month)	1.0844 (1.0677, 1.1014)	< 0.001*	1.0833 (1.0661,1.1007)	< 0.001*
	Severity ^a^	0.9597 (0.9462, 0.9734)	< 0.001*	0.9500 (0.9305,0.9699)	< 0.001*
	Log Dose MPH, (mg per kg)	2.2766 (1.6855,3.0750)	< 0.001*	2.3653 (1.6602,3.3699)	< 0.001*
	Taking risperidone	0.9035 (0.6720, 1.2150)	0.503	–	–
	Taking Fluoxetine	0.7398 (0.4524, 1.2097)	0.227	–	–
	ODD	0.6842 (0.4648, 1.0070)	0.064	–	–
	Mood disorder ^b^	0.6846 (0.2952,1.5873)	0.379	–	–
	Anxiety disorder ^c^	1.9619 (0.7667,1.5672)	0.614	–	–
	Other comorbidities ^d^	0.9692 (0.8631,1.0883)	0.587	–	–
Random intercept		–	Variance estimation		(95% CI)
		–	1.452		(1.2931, 1.6108)

^a^ The OR and its CI is computed for the centered value(i.e. severity- 51.3817). ^b^ Mood Disorder is the combination of Mood and bipolar disorders. ^c^ Anxiety disorder is a combination of GAD, SAD, OCD, Phobia, and Anxiety disorder. ^d^ Other comorbidities are included MR, LD, stutter, Tic, and MDD. *Significant at 0.05

In order to calculate the optimal dose, we use the coefficient of the final multivariable random effect model (Table 3).

**Table 3:** Final multivariable random effect model

	Covariates	Coefficient	(95% CI)	*P*-Value
Fixed effects	Intercept	1.9377	(0.9286, 2.9469)	< 0.001*
	Time interval (month)	0.0800	(0.0640,0.0959)	< 0.001*
	Severity	−0.0512	(−0.0720,-0.030)	< 0.001*
	Log Dose MPH, (mg per kg)	0.8610	(0.5069, 1.2149)	< 0.001*
Random intercept	Variance estimation		(95% CI)	
	1.452		(1.2931, 1.6108)	

*Significant at 0.05

**Fig. 1:**
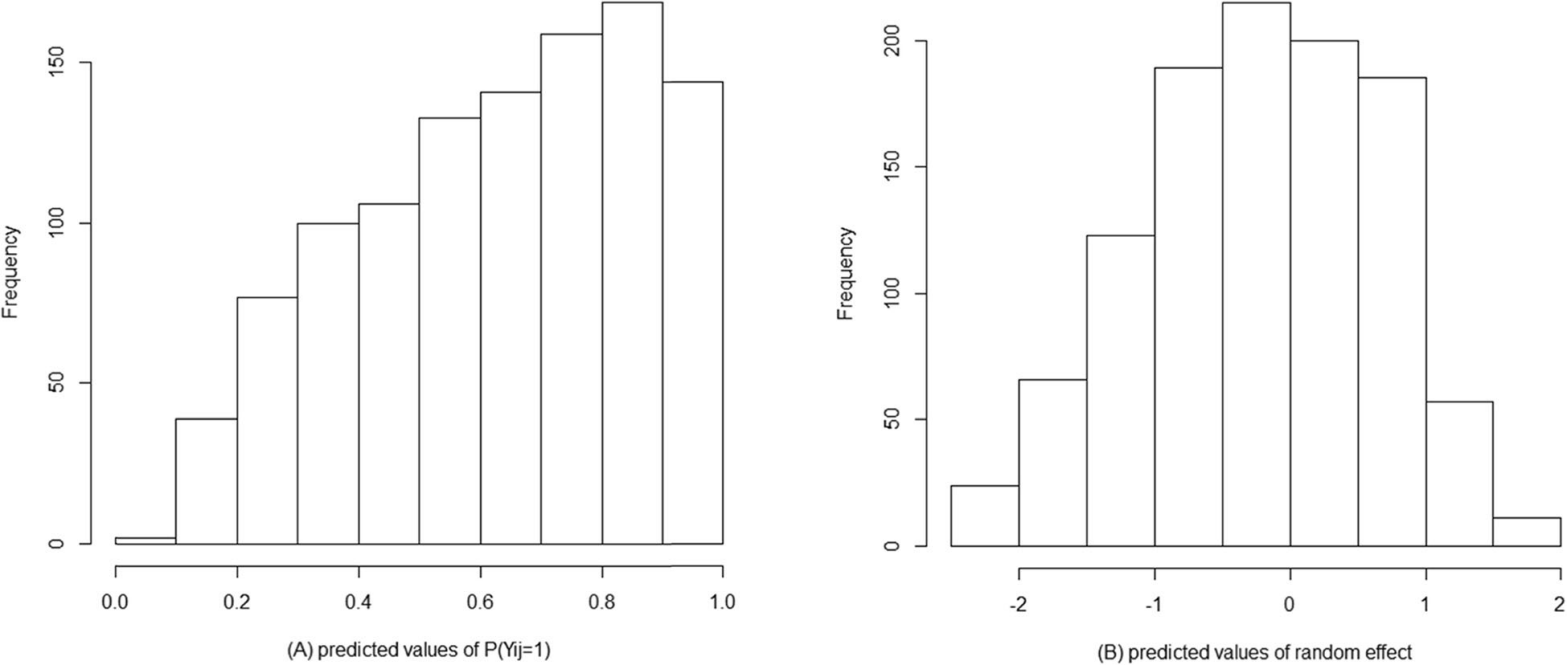
The histogram of (**a**) the predicted value for *P*(*Y*_*ij*_ = 1) at the last visit and (**b**) the predicted value for the random effect (${\hat{b}}_i$)

The target value for *P*(*Y*_*ij*_ = 1) is *p*_0_ =0.9866. The histogram of predicted values of P(Y_ij_ = 1) and random effect are shown in Fig. 1. To determining the dosage of MPH for a new patient (k) at the first visit, the initial dose can be calculated as follows:

4 $$ {D}_{k1}=\exp \left\{\frac{4.2990-1.9377+0.0512\times \left( severity-51.3817\right)-{\hat{b}}_{k,0}}{0.8610}\right\} $$

**Fig. 2:**
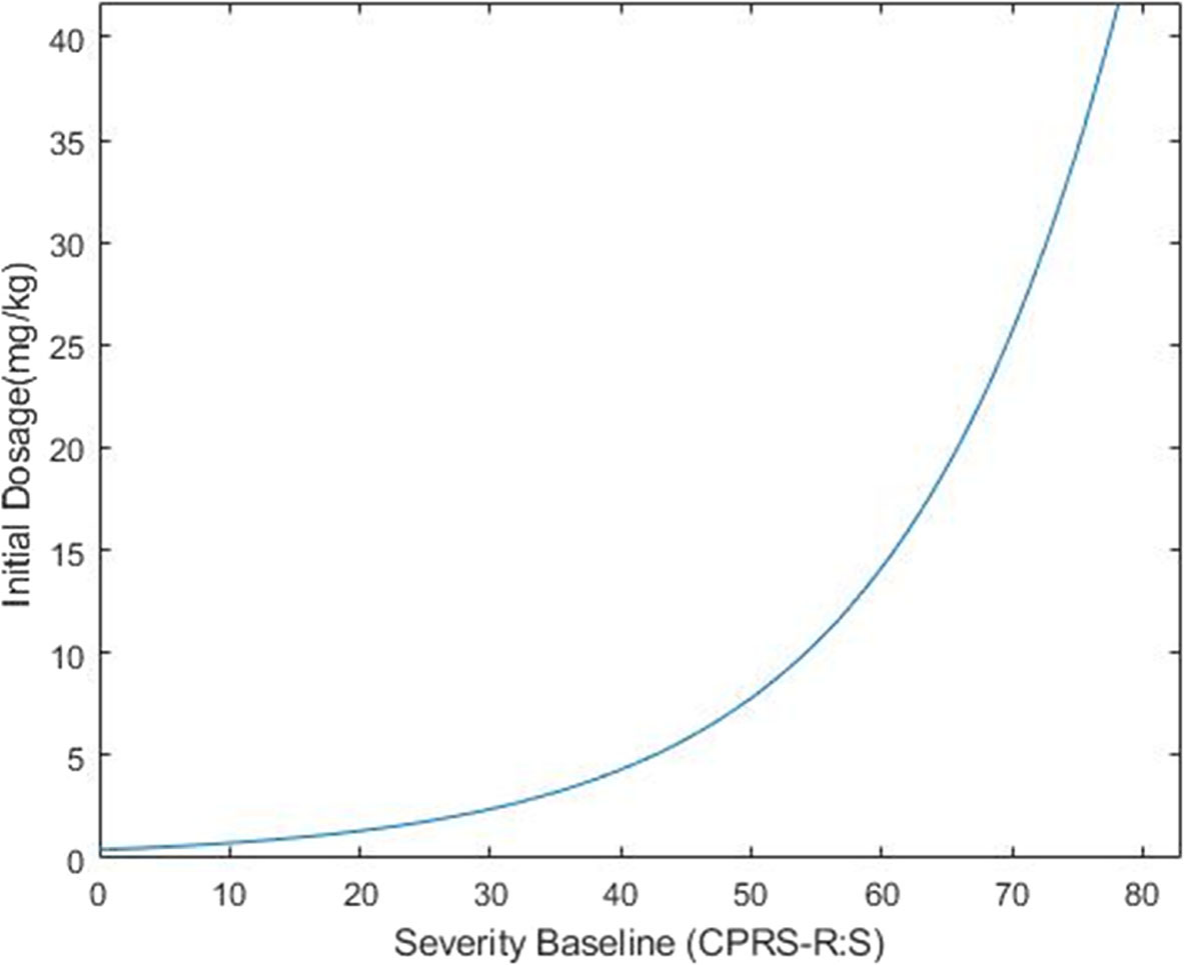
The optimum initial dosage of MPH in ADHD, based of severity of baseline (CPRS-R:S) The figure represents average patients

Where 4.2990 is the logit of the predicted value *p*_0_, 1.9377 is the estimate of fixed intercept, and 51.3817 is the mean of the severity of ADHD at baseline. The diagram of this equation is shown in Fig. 2.

Also at the n-th time point (follow-up), the dosage can be calculated based on the following formula:

5 $$ {D}_{kn}=\mathit{\exp}\left\{\frac{\mathrm{logit}\ \left({p}_0\right)-1.9377+0.0512\times \left( severity-51.3817\right)-0.0800\times time-{\hat{b}}_{k,n-1}}{0.8610}\right\} $$

**Fig. 3:**
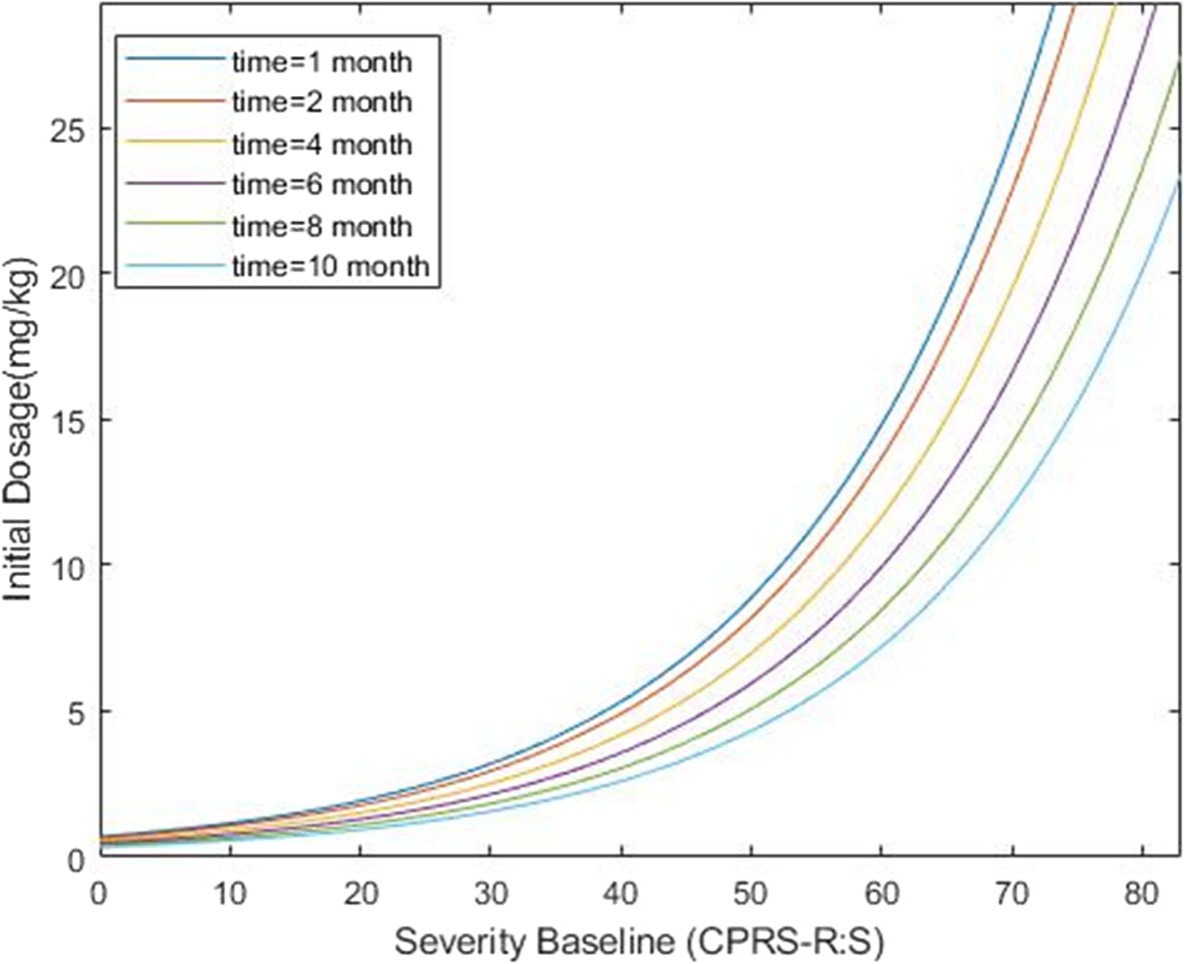
The optimum dosage of MPH in ADHD, based on severity of baseline for different time points. This figure corresponds to the average patients

Where *p*_0_ is the target probability of improvement, *severity* is the severity of ADHD at baseline (based on CPRS-R:S) and *time* is the number of months on treatment. The diagram of the dosage recommendation for different time points is shown in Fig. 3. In order to illustrate the above relationship, the optimum dosage is obtained for some hypothetical data (Table 4). Furthermore, the computer code for predicting b_i_ and for computing the optimal dosage is available in Additional file 1.

**Table 4:** Recommended dosage for hypothetical patients

Patient ID	weight	Severity of baseline	Time (month)	Baseline response	Dichotomized responses	${\hat{b}}_i$	Recommended dose (mg/kg)^a^
1	24	56	1	45	0	− 0.1647	0.1638
1	24	56	2	42	0,0	−0.1651	0.1247
1	24	56	4	39	0,0,1	−0.1690	0.0871
2	23	78	1	67	0	−0.1497	0.6023
2	23	78	2	65	0,0	−0.1449	0.4781
2	23	78	5	39	0,0,1	−0.1456	0.0773
2	23	78	6	30	0,0,1,1	−0.1439	0.0412

^a^: the recommended dose is predicted based on the formula (5)

## Discussion

In this study, the clinical severity at baseline, logarithm of dosage of MPH, and time duration of receiving the MPH were associated with improvement of ADHD.

The dosage of MPH in logarithmic scale had a meaningful association with improving from ADHD, in which the chance of improvement increased 2.36 times as one unit adding in the dosage of MPH in the logarithm scale.

According to multivariable random effect logistic model (Table 2), the odds ratio of severity is 0.9500, therefore with increasing one unit in severity, the chance of improvement decreased. Also, the odds ratio of time is 1.0833, therefore increasing one month receiving MPH, the chance of improvement increased.

As ADHD can cause social, emotional and economic failure and also increased mortality [22], pharmacological treatment in many cases is inevitable. On the other hand MPH as the most common drug, has an important complication, so trying to prescribe the optimum dose for each patient is a considerable achievement.

In this study, although the rate of males and females was not the same, as the demographic data were not different, we didn’t make subgroups of males and females to analyze the data.

We used generalized linear mixed models (GLMMs) method to personalize the dosage of MPH in ADHD. Generalized linear models (GLMs) are used to investigate and analyze the relationship between clinical, demographic, and genetic covariates on the response variable such as patient recovery. But these models aggregately study the relationships and characteristics of individuals. To investigate the relationships of the response with the unique characteristics of the studied subjects, another family of statistical models, usually called GLMMs (or random-effects (REs) linear models) is used. Using these models can be a valuable tool and a comprehensive conceptual framework for the development of personalized medicine [23].

The fact that GLMMs have the concepts that allow explaining patient populations as a whole (the fixed effects) and, simultaneously, concepts that allow describing patients as individuals (the REs) suggests that these models include the key ideas for providing PM with a precise statistical language [24, 25]. Thus, the variation of random components are not only due to a mathematical artifact control for patients’ heterogeneity but also the consequence of actual variation in the biological and environmental factors making humans develop as individuals [26]. Hence, both biological and statistical evidence supports the development of a methodological tool for PM based on GLMMs.

In this study we proposed a drug dosage individualization procedure than could be considered as an extension of the individualization algorithm proposed by Diaz et al. for continuous responses to dichotomous responses [25, 27].

Based on the result of this study, in order to determine the dosage of MPH for a new patient (initial dose), the greater the severity of baseline is, the higher the initial dosage is required.

To recommend the dosage in the next times, first, due to the information of the patient the random coefficient should be updated then the dosage should be calculated for him/her. According to the results of this study, in patients with the same severity of disease and same response to the treatment, with increasing the period of receiving MPH, the optimum dose of MPH decreased on the log scale by the rate of 0.08 mg/kg. Furthermore, the more the severity of ADHD is, the higher of the optimum dose is needed, i.e. considering that response to the treatment is similar among patients, at the same time the optimum dose of MPH increased by the rate of 0.06 mg/kg as the severity of ADHD based on CPRS-R:S increased. As seen in Fig. 1, for an average patient, the optimal dose with higher degree of severity is increased. Also, the optimal dose of MPH decreased when the time duration of receiving MPH increased.

To best of our knowledge, few studies on factors affecting treatment in ADHD have been published. In a Cochrane systematic review by Osland et al. in 2018, mentioned 3 studies that evaluated the MPH in ADHD [28]. In a cross-over trial, children were randomized to three weeks each of MPH, dextroamphetamine, and placebo. MPH significantly decreased hyperactivity at all doses [29]. In this study, they did not propose any optimum dose of MPH. Gadow KD et al. mentioned that treatment with 3 doses (0.1, 0.3 and 0.5 mg/kg) of MPH resulted in best improvement of ADHD with no significant differences between 0.1-mg/kg and 0.3-mg/kg dose on any of dependent measures [30].

In another study evaluating the treatment of ADHD in children with tic A, The dosage of average 25.9 mg/d of MPH was proposed for ADHD [31]. Therefore it seems that no study had been done to evaluate the effective factors to estimate the best dosage.

The strengths of this study include its conduct with a large and diverse sample of children with the main diagnosis of ADHD and the mean follow up period of 22.42 month. Also, we used the CPRS-R:S version of it because of its reliability and validity in the Persian version [20]. Furthermore, we used GLMM to individualize the dosage of MPH. Since participants were randomly recruited in this study across a referral hospital; therefore samples may not represent the population of ADHD children, hence it is proposed that a multicenter study could be done to better generalizability of the findings. However, full consideration of all factors that may have an impact on the improvement of ADHD, such as those related to the social economic status of children’s family, environmental factors like possible stresses that may be encountered by any patient, was outside the scope of this paper and hence these may represent confounding variables. Assessing factors associated with improvement for each comorbidity separately was also outside the scope of this paper. Nevertheless, the results of this study provide an individualized dosage for each patient due to factors affecting their improvement.

## Conclusions

In this paper, we propose a two-step procedure to make personalized dosage recommendations. The key idea of this method is to utilize subject-specific random effects from longitudinal responses specifying unique individual information that could contribute to post-treatment outcomes. In order to determine the dosage of MPH for a new patient, the greater the severity of baseline is, the higher the initial dose is required. Furthermore, in the next visits, to recommend the dose, the estimation of the random coefficients should be updated. The optimum dose increased when the severity of ADHD increased. Also, the results show that the optimum dose of MPH as one proceeds through the period of treatment, will decrease.

## Supplementary information

**Additional file 1.**

## Data Availability

The datasets used and/or analyzed during the current study are available from the corresponding author on reasonable request.

## Abbreviations

ADD: Attention deficit disorder
ADHD: Attention deficit hyperactivity disorder
CPRS-R:S: Conners’ Parent Rating Scale-revised Short Form
CRS: Conners’ Rating Scales
GAD: Generalized anxiety disorder
GLM: Generalized linear models
GLMM: Generalized linear mixed models
LD: Learning disorder
MDD: Major depressive disorder
MPH: Methylphenidate
MR: Mental retard
OCD: Obsessive-compulsive disorder
ODD: Oppositional defiant disorder
PM: Personalized medicine
Re: Random-effect
SAD: Social anxiety disorder
