# Supplementary material for: Individualizing the dosage of Methylphenidate in children with attention deficit hyperactivity disorder
Source: BMC Med Res Methodol. 2020 Mar 11;20:56. doi: 10.1186/s12874-020-00934-y (PMC7065304; doi:10.1186/s12874-020-00934-y)
Supplement: Supplementary file 1 — Additional file 1. [file 12874_2020_934_MOESM1_ESM.docx]

Additional file 1

library(Matrix)

library(lme4)

library(optimx)

library(dfoptim)

library(nloptr)

rm(list=ls())

#loading DATA

#FINAL MULTIVARIAE ANALYSIS

# random intercept logit model with time duratin recieving MPH, severity of ADHD (CPRS-R:S) and logarithm of dose of MPH

gmF <- glmer(y2 ~ time+ severity_c+ logDose_per_kg+ (1 | ID),data = dataADHD2, family = binomial, nAGQ=1)

summary(gmF)

se <- sqrt(diag(vcov(gmF)))

# table of estimates with 95% CI

(tabF <- cbind(Est = fixef(gmF), LL = fixef(gmF) - 1.96 * se, UL = fixef(gmF) + 1.96 * se))

tabF

#OR

exp(tabF)

####**************prediction***************************************

#predictin of logit(P(Yij=1))

logit_p1<-predict(gmF, newdata = dataADHD2, newparams = NULL,

re.form = NULL,

random.only=FALSE, terms = NULL,

type = c("link", "response"))

#histogram for prediction of logit(p(y=1))

hist(logit_p1,

main=NULL,

xlab="predicted values of logit(P(Yij=1))")

p1<-exp(logit_p1)/(1+exp(logit_p1))

#histogram for prediction of p(y=1)

hist(p1,

main=NULL,

xlab="predicted values of P(Yij=1)")

#prediction of random effect

p2<-predict(gmF, newdata = dataADHD2, newparams = NULL,

re.form = NULL,

random.only=TRUE, terms = NULL,

type = c("link", "response"))

#histogram for prediction of random effect

hist(p2,

main=NULL,

xlab="predicted values of random effect")

####**************prediction of dose***************************************

#please enter the severity of the disorder:

severity=56

sev= severity-51.3817

##prediction of initial dose for a new patient

pred_first_dose<-exp((4.2990-1.9377+0.0512*sev-p2)/0.8610)

summary(pred_first_dose)

##prediction of dose of MPH for a patient in t-th time point

##please enter the time:

t=2

pred_dose<-exp((logit_p1-1.9377+0.0512*sev-0.08*t-p2)/0.8610)

summary(pred_dose)
